# Supplementary material for: Are carbon emissions trading and green financial instruments synergistic? -Comprehensive quantitative research based on content analysis
Source: PLoS One. 2024 Mar 7;19(3):e0298601. doi: 10.1371/journal.pone.0298601 (PMC10919601; doi:10.1371/journal.pone.0298601)
Supplement: S1 Data — (ZIP) [file pone.0298601.s001.zip › Data/地级市Z_F工作报告.zip!/║╙─╧/─╧╤⌠╩╨/└┤╫╘╣1⁄2╓┌║┼í╛┬φ┐╦╩2╛▌═°í┐_2015.docx]

各位代表：

现在我代表市人民政府向大会报告工作请予审议并请各位政协委员和其他列席人员提出意见。

一、2014年工作回顾

一年来在省委、省政府和市委的坚强领导下我们认真贯彻党的十八大、十八届三中四中全会和习近平总书记系列重要讲话精神坚持“一统四抓三关键”总体部署全面落实市五届人大一次会议安排积极应对复杂严峻形势全力克服经济下行等困难统筹做好稳增长、调结构、强基础、促改革、惠民生各项工作经济社会继续保持较好发展态势。

(一)稳增长、保运行经济在困难中回升向好。面对年初全市经济低位开局、主要指标全面回落的困难局面我们把稳增长、防下行摆在更加突出的位置围绕困难点、潜力点、增长点长短结合分类施策精准发力全力推动经济企稳回升。制定实施稳运行保增长若干措施和“五个一百”行动计划强化经济运行督导推行“一企一策”针对性帮扶措施及时解决经济运行中的困难和问题。加大对金融机构的考核奖励力度提高市县担保机构资本额度推动银行、企业、担保公司三方合作预计全年新增贷款215亿元、融资总规模达到300亿元。实行差别化用地保障措施积极协调优惠电价重点项目和重点企业用地、用电得到较好保障。开展“项目建设提升年”活动完善项目建设协调推进机制300个省市重点项目建设扎实推进。二季度以来经济增长速度逐步回升、趋稳向好。初步预计全市生产总值增长8.5%规模以上工业增加值增长11.2%全社会固定资产投资增长19%社会消费品零售总额增长13%地方公共财政预算收入增长14.1%。

(二)抓大事、求突破重点工作扎实推进。坚持聚焦聚神聚力聚效以“三区”建设、新型城镇化、改革开放、环境建设及迎通水等重点突破带动整体工作提升。产业发展质量明显提升。深入实施“龙腾计划”、“雁阵计划”、“金地计划”谋划推进“一县一业”主导产业培育全市特色主导产业定位更加明晰4个产业集群纳入省重点培育的超百亿元产业集群。龙成重型钢构、富士康智能显示终端等一批重要工业项目开工建设河南油田特种蜡等一批重大项目前期工作扎实推进。预计产业集聚区固定资产投资1300亿元规模以上工业企业主营业务收入1900亿元对全市工业经济的贡献率超过60%超百亿元产业集聚区达到8个。15家企业进入全省民营企业100强总数居省辖市第一。服务业“两区”发展规划全部获批完成投资40亿元。老界岭·恐龙遗迹园成功创建国家5A级景区新增4A级景区3家旅游收入超过190亿元。西峡县跨入全国旅游标准化示范县行列。广发银行、洛阳银行南阳分行开业南阳银行改革重组并入中原银行。获批“全国中医药服务贸易先行先试重点区域城市”。现代物流、电子商务、信息消费等现代服务业发展提速。新建高标准粮田100万亩粮食总产实现十一连增达到102.4亿斤获得“全国粮食生产先进单位”称号。畜产品总量实现“五连增”。“中线渠首”有机农产品进入北京市场。农业产业化集群销售收入580亿元新增省级农业产业化龙头企业10家、农民合作社2953家、家庭农场603家、土地流转面积42万亩。新型城镇化步伐加快。预计城镇化率达到40.6%。出台实施中心城区“两度两力”提升行动计划。荣阳铝业、大商新玛特第四代购物中心等一批重要产业项目稳步推进。光武西路等5条道路建成通车18条城市道路有序实施外环路前期工作扎实开展。内河水系综合开发工程深入推进温凉河沿线房屋征迁任务基本完成、整治建设工程全面开工。白河滨水绿道全线贯通。“三馆一院”、市中心医院分院、市一高中新校区等一批公共服务设施即将开工。妥善处置“瘦身钢筋”事件集中开展专项整治房地产市场秩序趋于好转。国家卫生城市创建工作加快推进。数字城管项目基本完工。市城乡一体化示范区建设提速11条道路建成通车核心区路网基本形成王老吉等一批项目建成投产。县城建设步伐加快亮点纷呈。西峡县入选全省10个新型城镇化试点县。19个镇进入全国重点镇行列9个镇入选全省新型城镇化试点镇。美丽乡村建设有序推进农村人居环境不断改善。各项改革全面深化。研究出台推进重点领域关键环节改革决定。市县政府职能转变和机构改革有序推进食品药品监管体制改革稳步实施财税体制、事业单位分类改革深入推进。新取消、下放、合并行政审批事项56项。全面推行工商登记便利化改革新增市场主体6.3万余户增幅居全省第一位 侯卫东官场笔记。投资体制改革积极推进企业投资备案效率提高40%。牧原股份:上市中源化学借壳重组三利热能等3家企业成功挂牌。农村土地承包经营权确权登记试点工作基本完成现代农业综合改革试点工作取得阶段性成果。开放招商成效明显。成功举办第十一届玉雕节暨国际玉文化博览会、月季花展和第二届仲景论坛。“人才回归、全民创业”战略深入实施引进回创企业814个、高层次回归人才147人。京宛对口协作持续深化3个结对区县签订帮扶协议争取对口协作资金1.6亿元。中关村南阳科技园入驻项目12个京宛科技综合服务平台建设完工水科技产业园、水博园等项目进展顺利。开放平台建设取得重大突破南阳卧龙综合保税区获国务院批复成为全省第二个综合保税区。全年新签约页岩气装备等262个亿元项目其中宇阳新能源材料园等209个项目开工建设。实施与央企合作项目37个建成12个。预计实际利用外资6.7亿美元、增长3.3%到位省外资金538亿元、增长12.5%进出口总值19.7亿美元、增长10.2%。国宇密封公司成功收购德国威尔伯特公司。与意大利阿斯蒂市结为友好城市。生态环境建设力度加大。保护“母亲河”行动强力推进白河及鸭河口水库水生态得到有效恢复。入库、出省河流水质实现在线监控集中式饮用水源地水质全部达标设立国家首个重点流域面源污染综合治理试验区大气环境质量监测数据实时发布节能减排完成省定目标。高标准完成南水北调中线干渠、重要通道绿化任务完成营造林140.3万亩。晋升国家级自然保护区1个、森林(湿地)公园3个。跻身国家首批生态文明先行示范区入选第二批全国水生态文明城市建设试点。打赢迎通水攻坚战。开展两个“百日会战”和集中冲刺实施库区及上游水污染防治和水土保持项目100多个建成污水处理厂26个、垃圾处理场31个拆除养鱼网箱4万余个丹江口水库水质稳定保持在Ⅱ类标准施工环境保障有力干渠及配套工程建设基本完工移民帮扶力度加大移民群众收入较搬迁前实现翻番。经过全市上下合力攻坚、艰苦奋战南水北调中线顺利通水一渠清水送京津的世纪梦想成为现实再次书写了忠诚奉献、大爱报国的南阳担当!

(三)打基础、创优势发展支撑能力进一步增强。坚持远近结合着力打基础、强支撑、创优势、增后劲。综合交通体系加快构建。宁西铁路二线进入全面施工高潮。蒙华铁路控制性工程开工。郑万铁路即将开工。加密南阳至郑州、北京、广州的航班新增大连至南阳到昆明航班南阳飞行训练基地前期工作进展顺利。三淅高速建设快速推进。农村公路三年行动计划乡村通畅工程启动实施建成县乡公路450公里、村道1000公里、桥梁6500延米。能源建设成效明显。天池抽水蓄能电站附属工程和内乡煤电运一体化项目开工建设。南阳南输变电站加快建设南阳中输变电站即将开工。2个风电项目获省核准8个光伏发电项目前期进展顺利。创新能力稳步提高。109个科技创新项目有序实施。成功申报国家、省重大科技计划项目50个新认定国家高新技术企业11家。创建院士工作站2个获国家专利1270件。信息化建设步伐加快。出台实施智慧城市建设规划纲要“南阳国家级智慧政务建设试点示范城市”战略合作协议签约4G覆盖范围持续扩大智慧城市云数据中心建成投用。水利基础设施建设扎实推进。实施18个中小河流治理项目鸭河口灌区续建配套节水改造项目即将完工120座小型病险水库除险加固顺利完成。

(四)惠民生、保稳定社会建设水平持续提升。加大投入力度持续实施十项重点民生工程预计财政民生支出343亿元增长12.1%占财政总支出的76%。新增城镇就业7.06万人新增农村劳动力转移就业9.28万人。预计城镇居民人均可支配收入增长10%农民人均纯收入增长12%。社会保障水平进一步提高各项社会保险参保人数达到823.3万人次。城乡社会救助体系逐步完善48.9万城乡低保人口实现应保尽保。保障性住房开工建设4万余套、基本建成2万余套。新解决66.9万农村居民饮水安全问题11.5万农村贫困人口稳定脱贫。着力解决中心城区“上学难”、“大班额”问题制定实施中小学建设方案14所学校开工建设。高招一本上线率居全省第二位。市职教园区即将开工建设。南阳师范学院和南阳理工学院进入全省第二批整体转型发展大学行列。文化惠民工程深入实施免费开放博物馆、纪念馆达到16家县区公共电子阅览室建成投用。“唱响白河”等群众文化活动受到好评。居民健康卡发放工作经验在全国推广医联体、“先诊疗后结算”等工作走在全省前列。新农合大病保险制度和县级公立医院综合改革启动实施。实施单独二孩生育政策人口出生率预计控制在12.5%。以内。全民健身示范市建设深入推进成功举办第七届全省少数民族运动会。老龄、妇女儿童、残疾人、慈善等事业全面发展。信访稳定工作扎实有效法律援助覆盖面不断扩大。食品安全和安全生产形势总体平稳。社会治安综合治理深入推进打击刑事犯罪成效突出现行命案全部侦破反恐防恐工作更加主动重点部位防恐形势好转社会大局安定有序。

一年来我们深入开展党的群众路线教育实践活动工作作风明显转变自觉接受人大和政协监督办理市人大代表建议218件、市政协提案676件。“一改双优”、“三亮三评”活动深入开展行政审批服务、公共资源交易、便民服务“三个中心”建设取得良好成效市长热线服务平台资源整合、功能拓展政府效能和服务水平得到提升。依法行政深入推进反腐倡廉工作取得新成效。精神文明建设进一步加强公民道德素质和社会文明程度全面提升。国防教育和国防后备力量建设持续强化驻宛解放军、武警部队和民兵预备役人员为地方发展作出新贡献。工商、税务、海关、检验检疫、审计、统计、史志、档案、民族、宗教、外事侨务、邮政、通讯、电力、农机、气象、地震、人防、对台、驻外办事机构等工作都取得了新成绩。

各位代表!这些成绩的取得是省委、省政府和市委正确领导的结果是市人大、市政协大力支持的结果是全市人民共同奋斗的结果。在此我代表市人民政府向全市广大干部群众向人大代表、政协委员、各民主党派、无党派人士、工商联、各人民团体、离退休老同志向驻宛部队、武警官兵、公安民警向中央、省驻宛单位向所有关心支持南阳发展的各界朋友表示崇高的敬意和衷心的感谢!

肯定成绩的同时我们也清醒地看到经济社会发展中还存在一些突出矛盾和问题一是发展速度还不够快经济增速低于预期目标经济持续向好的基础还不稳固部分行业企业经营比较困难大个支撑项目少发展后劲不足。二是结构性深层次矛盾还比较突出农业基础依然薄弱工业大而不强特色主导产业不突出服务业规模小。城镇化率偏低中心城区带动作用弱。三是公共产品和公共服务欠账较多一些涉及群众切身利益的就业、就学、就医、环保、拆迁等问题尚未得到根本解决改善民生和加强社会治理任务依然繁重。四是内河水系治理等重点工作由于前期准备不足推进协调中出现新情况工作任务没能按预期节点完成。五是政府职能转变还不到位体制机制障碍多项目审批难、耗时长问题尚未有效解决政府系统一些领导干部及工作人员能力不强、作风不实、服务不优、效能不高、为政不廉等问题仍不同程度存在。对此我们一定要高度重视认真加以解决。二、2015年工作总体要求和目标任务

南阳同全国、全省一样也步入经济发展新常态发展环境发生新变化总的来看是机遇挑战并存机遇大于挑战。从机遇看我国经济发展总体持续向好全面深化改革将释放新的动力活力“一带一路”、京津冀协同发展、长江经济带三大国家战略深入实施南水北调中线通水更有利于我市深化与京津地区合作、对接武汉西安等发达地区新常态下产业转移趋势不会改变新业态、新技术发展迅猛为我市承接产业转移、抢占新的产业发展制高点创造了有利条件郑万铁路、宁西铁路二线、蒙华铁路、天池抽水蓄能电站、内乡煤电运一体化等重大项目开工建设综合保税区获批既利于增强投资拉动、促进经济增长又利于提升综合承载保障能力为长远发展增创新优势。从挑战看全国经济增速换挡下行压力依然较大和周边先进地区差距拉大面临着不进则退、慢进亦退的竞争压力我市传统产业比重大、新兴产业规模小培育新的增长点更加迫切。因此我们既要把握机遇坚定信心又要保持清醒积极应对挑战、化解风险牢牢把握新常态下经济工作主动权努力在新一轮发展中抢得先机争取主动。

今年政府工作的总体要求是认真贯彻党的十八大、十八届三中四中全会、中央经济工作会议精神全面落实省委九届八次全会和经济工作会议部署以适应新常态、抢抓新机遇、实现新跨越为主旋律以项目为抓手坚持“一统四抓三关键”总体部署坚持“一优一高一稳定”工作方针实施提升中心城区“两度两力”和富民强县“两轮驱动”加快推进经济、社会、生态“三重转型”奋力打造大美南阳、活力南阳、幸福南阳为全面建成小康社会蓄势加力。

本着积极可行原则根据我市实际情况结合当前宏观经济形势、“十二五”规划和与全省同步全面建成小康社会要求今年全市经济社会发展的主要预期目标是生产总值增长8.5%规模以上工业增加值增长11.5%地方公共财政预算收入增长11%全社会固定资产投资增长19%社会消费品零售总额增长13%城镇居民人均可支配收入和农民人均纯收入均增长9%以上居民消费价格涨幅3%左右节能减排和环境质量改善完成省下达任务。

实现上述目标做好今年政府工作要把握好以下几点一是转变观念促转型。进一步解放思想更新观念破除盆地意识和路径依赖加快发展观念、工作方式方法转变坚持“一优一高一稳定”调中求进努力做到“转”和“赶”的有机统一。二是板块突破强支撑。以市城乡一体化示范区、综合保税区、产业集聚区、商务中心区、特色商业区、都市生态农业区等载体平台为核心聚力打造汇聚要素、支撑增长的经济集聚板块构筑多区支撑经济发展的新格局。三是补短抓新增动力。以开放招商为总路径紧盯工业、服务业等薄弱环节补齐发展短板增强内生动力抓住新兴产业发展机遇积极发展新业态、新模式壮大新的接续力量。四是改革创新破难题。用改革的精神、创新的手段破除制约发展的体制机制性障碍大力推进科技创新、机制创新、管理创新以改革促创新以创新解好扣不断增强发展动力。五是聚焦聚神求实效。把力量聚焦到发展上、聚焦到项目上强力实施“113215”投资促进计划以项目建设的新突破支撑南阳发展的新跨越。

重点抓好以下九个方面的工作

(一)聚焦特色主导产业培育推动工业转型发展迈出新步伐

围绕做强工业这一战略任务按照“市有千亿、县有百亿产业集群”的目标狠抓大载体、大企业、大项目推动工业加速转型升级、做大做强。一是强力推进产业集聚区建设和特色产业集群培育。把我市基础较好的食品加工业率先培育成千亿元产业。着力培育先进装备制造、光电电子信息、汽车及零部件、生物医药、纺织服装、新能源六个特色主导产业实行“八个一”工作推进机制强力组织实施确保今年有大的突破。突出“一县一业”实施百亿元特色产业集群培育工程力争县区超百亿元和超50亿元的产业集群分别达到2个和10个。加快产业集聚区建设力争全市产业集聚区固定资产投资突破1500亿元规模以上企业主营业务收入突破2000亿元努力推动新能源、淅川产业集聚区晋升为二星级光电、社旗、内乡、镇平产业集聚区晋升为一星级。推进产业集聚区与所在乡镇行政区域管理套合。支持官庄石化园区等专业园区晋升为省定产业集聚区。二是推动传统产业转型升级。对冶金、建材、化工等传统产业综合运用延伸链条、技术更新、兼并重组等方式发展下游产品和消费终端产品探索新的经营模式和发展路子实现转型脱困。推动装备制造智能化、集成化引导制造企业从单纯生产产品向提供软件、服务和整体解决方案等集成业务转变。三是培育壮大骨干企业。实施大企业大集团培育工程围绕80家高成长性骨干企业出台发展规划和扶持政策力争主营业务收入超百亿元的企业达到4家超50亿元的企业达到5家超30亿元的企业达到8家超10亿元的企业达到20家。强力推进企业上市力争张仲景大厨房、全宇制药、西施兰等3～5家企业上市挂牌石油二机、森霸光电、宛西制药3家企业进入上市辅导乐凯华光、福森药业等6家企业完成股份制改造。四是狠抓项目建设。实施亿元以上工业项目352个年度完成投资470亿元。重抓重推河南油田特种蜡、南阳烟厂异地技改搬迁、荣阳铝业、北新建材等30个重大工业项目实行专题专案专班推进年度完成投资100亿元以上。鼓励支持企业技术改造重点实施汉冶特钢精轧机改造等96个技术改造项目年度完成投资70亿元。对年度技改设备投资超5000万元的企业给予贴息支持。扎实推进城区老工业区搬迁改造项目。争取国家支持资源枯竭型工矿区发展政策。五是强化服务保障。强化市级领导联系分包重点产业、重点企业、重点项目制度。建立工业经济协调服务周例会制度。研究解决中心城区重点工业企业土地证、房产证办理问题。开展校企、银企、产销对接活动。探索设立产业投资基金。组建南阳担保集团设立企业缓释基金。

(二)以“两区”建设为重点推动服务业发展实现新突破

以服务业“两区”和各类专业园区为载体以我市特色优势产业为主攻方向滚动实施100个服务业重点项目加速培育壮大优势服务业集群打造高成长性服务业大市形成支撑经济发展的新动力(310328,基金吧)。一是加快推进“两区”建设。把服务业“两区”建设摆上重要日程确保今年见成效、出形象。一季度全面完成“两区”空间规划和控制性规划编制报批工作。强力推进市商务中心区建设实现基础设施基本到位确保今年整体上有大起色、大突破。加快卧龙岗、张仲景两个特色商业区建设破解拆迁和融资难题确保今年有实质性进展。推动镇平、社旗、内乡、西峡、淅川等基础条件较好的特色商业区进一步突出特色、提升功能桐柏、南召、唐河、新野、方城等基础条件较为薄弱的特色商业区扎实起步。坚持基础建设和招商引资同步推进利用京宛合作、沪宛合作等平台瞄准龙头企业、新型业态引进实施华南城等重大项目每个“两区”至少有1个千万元以上项目签约落地。二是突出抓好重点产业发展。大力发展文化、旅游业。把文化、旅游业作为南阳的支柱产业来培育打造。加快推进各类文化产业园区建设。引进、培育文化创意、动漫、软件、大型演艺等新兴业态。围绕增量抓品牌、存量抓提升推动旅游业十大项目建设打造南阳十大精品旅游目的地创建和提升10个A级景区打造和推出10个乡村旅游示范村实施旅游四项提升工程。聚焦北京市场加大旅游宣传推介力度。提升商贸、物流业发展层次。围绕建设区域性商贸、物流中心筛选确定50家商贸流通企业进行重点培育引进实施一批具有区域性贸易中心功能的大项目引进大型物流企业对我市物流资源进行整合重组实施中心城区商业网点规划加快推进内乡煤电运一体化等项目建设。推动商业模式创新大力发展电子商务出台发展规划、三年行动计划和扶持政策支持成立网络营销协会建设人才培训基地、电商孵化基地和实训基地鼓励企业建立网络营销平台推动淘宝“特色中国南阳馆”和慧聪网“玉文化产业带”尽快上线运行加快中关村科技园电子商务仓储物流园建设力争南阳电子商务创业基地创建省级电子商务示范基地鼓励有条件的企业争创电子商务示范企业。积极发展金融服务业。完善金融体系再引进2家金融机构探索组建农村商业银行推动保险、证券、信托等金融机构来宛设立分支机构积极引进设立各类投资基金规范发展市、县投融资平台。鼓励金融机构创新金融产品推动企业更新观念通过发行债券、股权交易等新模式实现直接融资。谋划推动农村金融改革。优化金融生态环境严厉打击非法集资确保不发生金融风险。培育壮大健康服务业。以建设国内知名品牌仲景健康服务业基地为目标制定健康服务业发展行动计划大力发展以中医药养生为特色的健康服务业加快推进海王生命健康产业园等重点项目建设。推动中医药贸易和养老服务业发展。规范有序发展房地产业。以保障合理的市场需求为导向推动房地产业规范有序发展引进知名房地产企业进入南阳市场解决结构性供需矛盾防范化解市场风险提升房地产业发展水平。积极培育文化旅游地产、养老地产等新兴业态。三是强化服务业发展支撑保障。理顺管理体制整合加强服务业发展机构和力量。制定并严格落实支持服务业发展的专项配套政策。降低医疗卫生、教育培训、健康养老等领域准入门槛。

(三)围绕稳粮、转型、增效推动农业发展走出新路子

以发展现代农业、生产有机产品为方向以有机农产品进京和创建国家出口农产品质量安全示范市为引领把大农业做成大特色把大特色做成大产业实现稳粮保粮和富民强市双赢。一是提高粮食综合生产能力。实施高标准粮田“百千万”工程全年建成高标准粮田90万亩持续改善农业生产条件粮食总产量保持在100亿斤以上。二是加快建设农业产业化集群。抓好20个重点集群建设着力培育壮大科尔沁肉牛、牧原生猪、赊店酒业有机粮食三大产业集群全市集群收入增长15%以上。三是抓好有机农产品生产。以渠首有机农产品示范基地建设为引领实施农业标准化工程加快培育具有地域特色的优质品牌农产品推动畜牧养殖与高效农业融合、循环发展全年新发展有机农产品基地50万亩新增“三品一标”认证面积30万亩。建设环丹江口库区有机特色林果示范区。强力推进有机农产品进京。积极推进中原有机农产品贸易加工区规划建设。四是大力发展都市生态农业。编制实施都市生态农业发展规划加强扶持引导创新农业业态加快基地建设打造近郊休闲观光圈、远郊绿色产业圈、山区生态涵养圈构建集高效产业、农产品物流加工、景观绿化、生态涵养、休闲观光等功能于一体的都市生态农业体系。五是加快构建新型农业经营体系。大力培育新型农业经营主体培养高素质的职业农民全年新发展家庭农场500家、农民专业合作社500家。完善土地流转公共服务体系新增土地流转面积100万亩。加快农业科技创新和重大技术推广。全面提升农业社会化服务水平。

(四)加强支撑能力建设为长远发展培育新优势

全面加强基础设施和人才队伍建设促进基础设施互联互通提高人才队伍质量增强支撑保障能力推动局部优势向综合优势转变。加快构建综合交通体系。大力推进宁西铁路二线、郑万铁路、蒙华铁路“三铁”建设全力服务保障施工环境力争完成投资78亿元。实现三淅高速南阳段建成通车、内邓高速通车运营。加快推进续建和6条新开工的国道、省道建设开工建设中心城区外环路东环路、南阳至官庄快速通道。新改建县乡公路450公里、村道1000公里、桥梁8000延米。增开南阳机场航线航班开工建设南阳飞行训练基地项目。积极争取宁西铁路客运专线进入国家中长期铁路建设规划开展南阳到济宁铁路前期工作。力争开工建设栾西高速公路和2个高速公路开口设站项目及南阳至鸭河快速通道。完成南阳至社旗快速通道前期工作。争取将方枣高速等2条高速公路纳入省规划加快推进周南高速南阳段前期工作启动南阳至渠首高速前期工作。积极推进唐河复航等项目前期工作。加快能源项目建设。强力推进“两电一气”项目完成投资40亿元以上。实现唐河石柱山、社旗风电项目6月底前并网发电开工建设4个风电项目。完善电网结构抓好南阳南、南阳中输变电站建设。再争取4个风电项目列入今年风电核准计划积极争取光伏发电补贴指标。加快推进鸭河电厂三期等重点项目前期工作 完美世界小说。加快信息网络系统建设。全面实施南阳智慧城市规划加大“两网一站”工作力度积极申报国家智慧城市试点。扩大4G和高速无线宽带网络覆盖面。积极推进三网融合完成有线电视网络双向化、宽带化升级改造。扎实推进国家信息中心南阳智慧政务示范市建设。加快水利基础设施建设。完成鸭灌、宋岗等大型灌区续建配套与节水改造年度任务搞好丹江口库区及上游重点地区水土保持二期工程建设。争取完成80座小水库除险加固和9条中小河流治理项目建设任务开工建设内乡北湾水库。再完成一批农村安全饮水工程。加快人才队伍建设。围绕经济社会发展需求坚持培养和引入并举深入推进人才回归战略、全民技能振兴工程、职业教育攻坚二期工程、高等教育内涵提升工程着力培养一批产业急需的技能人才大力引进一批高层次人才。高度重视企业家培育培养造就一批具有国际眼光、开拓精神、管理能力和社会责任感的商界“宛军”。

(五)以宜居宜业为方向推动城乡建设展现新面貌

实施提升中心城区“两度两力”和富民强县“两轮驱动”建设生态良好、产业兴旺、生活舒适的美丽城乡让宜居宜业成为南阳新名片。

强化规划引领。树立先进规划理念完善规划体系抓好中心城区郊区总体规划和示范区、内河水系、路网绿道、名人雕塑等建设急需的专项规划编制完善中心镇镇域规划完成新农村建设规划编制。启动城市轨道交通规划研究工作。继续推进各级规划展示设施建设逐步消除空白。

打造魅力宜居中心城区。以国家卫生城市和全国文明城市创建为抓手推进以产立城、以水兴城、以文塑城、以绿靓城建设宜居先行区。夯实宜居基础。围绕补足短板、完善功能、修复生态、增强承载加快路、水、气、热等基础设施建设完善教、医、文、体、娱等公共服务设施持续推进内河治理增加城市绿地和公共休闲空间让居民生活出行更便捷让清水绿树绕城来。着力解决城区交通拥堵问题建设联通顺畅城市路网以打通断头路、疏通卡脖子点为重点续建滨河路下延、白河大道下延和新建北京路南延、两相东路等城市道路。开工建设公交场站2个、客运场站3个完善交通标志优化公共交通线网新增公交车200台。推进南水北调麒麟水厂建设新建污水处理厂2座铺设供气管网97公里、供热管网14.6公里启动固废综合处理生态产业园区筹建工作再新建一批公厕。加快“三馆一院”、市中心医院分院等公共服务设施建设做好文博景区改造提升。推进白河城区段清淤和两岸景观改造提升上半年完成温凉河治理建设任务实施梅溪河、三里河截污治污工程。实施“一环四带六园六线九山”森林生态防护工程完成李宁体育园提升改造推进申伯公园等城区公园、绿地建设抓好道路、重要通道及入市口绿化提升加快紫山植物园、动物园建设完善提升独山、兰湖森林公园。继续实施“优美花城行动”。稳步实施城中村和旧城改造。推进板块发展。按照板块突破思路抓好多个经济板块发展。依托5个产业集聚区(工区)和4个装备制造园集中打造千亿级产业集群工业板块。推进高新区板块上档升级。抓紧推进商务中心区、卧龙岗、张仲景文化产业集聚区和南都古城建设尽快形成各具特色的服务业“隆起”板块。高起点快速推进综保区板块建设切实抓好职教园区板块建设。推动都市生态农业板块建设加快实施“两园一带”示范区等一批项目。加强城市管理。强化规划、国土、建设联合执法保持打击“双违”高压态势坚决不让一起新的违法建筑落地。推行精细化、规范化、数字化管理开展市容市貌、交通秩序等专项整治努力营造绿、亮、美、净、畅的宜居环境。全面加快市城乡一体化示范区建设。加快基础设施和产业项目建设争取早出形象、早见成效。掀起基础设施建设高潮力争15条道路全部建成通车加快10个村庄城镇化改造。推动产业起步全力推进商务中心区的金融集聚区和总部经济区建设加快实施王老吉二期等一批工业项目。加大招商力度尽快落地一批好项目、大项目。

提高县域宜居水平。强化县域多点支撑作用加快西(峡)-淅(川)、唐河、方城、镇平四个增长极建设大力发展南召、新野、内乡、社旗、桐柏五个节点县城推进唐河发展成50万以上人口城市其他县城向30万以上人口城市迈进。加强供排水供热供气和污水、垃圾处理等基础设施建设推进产业集聚区基础设施与县城衔接配套完善产业集聚区各类公共服务和生活配套设施增强县城产业和人口集聚能力。有重点地发展小城镇加快推进9个省级重点示范镇建设。分类推进新农村建设深入开展农村人居环境整治和美丽乡村建设。

创新新型城镇化推进体制机制。制定城镇基本公共服务常住人口全覆盖、户籍改革等配套措施确保农业转移人口进得来、落得下。积极争取省新型城镇化发展母基金探索建立城镇化发展子基金推行公私合营(PPP)城建模式。实行“五挂钩”用地考核评价机制积极稳妥推进人地挂钩试点提高城镇建设用地效率。

(六)提升公共服务水平在更高水平保障民生上实现新作为

保障和改善民生是政府一切工作的出发点和落脚点越是在困难条件下越要把改善民生放在更加重要的位置多做雪中送炭的事集中财力办好群众看得见、摸得着、能受益的事努力把人民的期待变成我们的行动把人民的希望变成生活的现实。一是千方百计扩大就业。鼓励支持企业吸纳就业。强化对高校毕业生、农村转移劳动力、城镇困难人员等重点群体的就业服务力争新增城镇就业6万人、农村劳动力转移就业6万人。二是提高社会保障水平。实施全民参保登记计划和扩面征缴行动。实现城乡居民与城镇职工之间养老保险关系可转移接续。新农合人均筹资水平提高到450元。启动实施城镇居民大病保险。全面推行社会保障一卡通。稳步推进机关事业单位养老保险制度改革。继续做好老龄工作。开展好社会救助工作加快建设一批社会福利中心。新开工保障性住房2.1万套。妥善解决农运会涉迁群众安置遗留问题。三是做好扶贫开发和移民工作。强力实施“两山一岗”扶贫工程把区域开发与精准扶贫结合起来稳定解决10.5万农村贫困人口脱贫。持续做好移民后续稳定发展工作推动移民发展致富把移民村率先建成美丽乡村。四是全面发展各项社会事业。办好人民满意的教育。实施第二期学前教育三年行动计划。强力推进义务教育均衡发展深入实施全面改薄计划和扩充城镇义务教育资源五年计划。持续用力解决“上学难”、“大班额”问题中心城区再新建改扩建中小学10所新建一批幼儿园。加快推进市一高中新校区建设。大力发展职业教育全面推进市职教园区建设开工建设南阳高级技工学校和南阳工业学校加快南阳农业职业学院二期工程建设步伐。支持南阳医专升格更名为张仲景医学院支持南阳师范学院、南阳理工学院示范性应用技术类本科院校试点建设。研究出台支持社会力量办学政策鼓励社会办学统筹规划加快教育事业发展。提高人民健康水平。推动医疗卫生工作重心下移、医疗卫生资源下沉进一步加强城乡医疗卫生服务体系建设解决好基层群众看病难、看病贵问题。提高基本公共卫生服务均等化水平。继续实施智慧卫生工程。大力发展文化、体育事业。继续实施文化惠民工程。加强文化遗产保护与合理利用。持续推进全民健身示范市建设推动体育事业和体育产业协调发展。加强场馆综合利用提升综合收益。整合基层文化、体育等资源积极推动基层综合公共文化服务中心建设。五是创新和加强社会治理。加快信息化在社会管理、便民服务等领域的应用。畅通群众诉求反映渠道强力推进市长热线扩容升级工程。启动实施50个中心城区社区信息化平台建设。深入推进依法治市做好普法教育工作提升法律援助保障水平。做好安全生产工作。推进食品药品监管体制改革全力保障食品药品安全。提高重大突发事件快速应对和处置能力。牢牢把握防暴恐、保稳定工作主动权深入开展打黑除恶行动防范和遏制危害人民群众生命财产安全的重特大治安事件坚决避免群死群伤重大事故努力创造和谐安定的社会环境。

(七)坚持标本兼治推动生态文明建设取得新成效

顺应人民群众对良好生态环境的期待以国家生态文明先行示范区建设为抓手坚持源头严防、过程严管、后果严惩治标治本多管齐下努力让人民群众喝上更干净的水、呼吸更清洁的空气在良好的生态环境中工作生活让美丽与发展同行。一是强化主体功能区战略。编制实施主体功能区规划按照重点开发区、限制开发区和禁止开发区划分要求加强空间管制优化空间布局科学划定耕地、水资源、生态控制线严格保护每一寸耕地、每一条河流、每一块林地。二是加强丹江口库区水质保护。加快“十二五”防治规划项目实施发挥好渠首水质监测应急中心作用全面推进重点流域面源污染综合治理试验区建设决不让任何污染项目在水源保护区落地坚决完成保护水质安全这一政治任务。三是推进生态建设工程。按照“三区三带”生态建设布局推进重点区域绿化造林完成营造林144万亩。着力实施南水北调中线工程渠首及干渠生态治理、石漠化治理等工程。再申报一批新的森林(湿地)公园。加快推进水生态文明城市建设试点工作。四是加强环境保护。全面实施蓝天工程加快推进工业脱硫脱硝等关键环节和蒲山等重点区域专项治理突出城市面源污染治理不断改善大气环境质量。持续推进碧水工程开展丹江口水库、鸭河口水库和白河沿线环境整治持续开展保护母亲河行动加快内河治理步伐对唐河、淮河等重点河流实施一河一策综合治理。实施乡村清洁工程全面改善农村人居环境。五是抓好节能减排。深入开展重点减排项目建设推进年活动。推进万家企业节能低碳行动实施一批节能重点工程和资源节约、循环经济示范项目。六是加强生态文明制度建设。健全环境损害鉴定评估机制和责任追究机制。加强制度创新力争在生态补偿机制、区域协调机制等方面实现突破。(八)建平台、招大商打造对外开放新高地

突出大招商、招大商深入开展开放招商年活动用好综保区平台抢抓产业转移机遇再掀开放招商新热潮。一是强力推进综合保税区建设。

把综合保税区作为发展外向型经济、提升对外开放水平和有效招商引资的重要载体作为对接郑州航空港经济综合实验区、融入“一带一路”的重要平台抢抓机遇统筹运作打造高端开放平台增创区域竞争新优势。对照封关验收的标准健全机构设置加快基础设施和监管、检验检疫等配套设施建设完成投资10亿元以上。加强产业谋划以消费类电子和智能终端为重点引进一批质量高、规模大的外向型产业项目。持续打造好高新区、经济技术开发区、各类产业集聚区等开放招商平台。二是提升招商引资工作水平。围绕主导产业突出大型劳动密集型代工企业、整体转移型产业集群、战略布局型产业和新业态、新模式等招商重点瞄准重点区域紧盯目标企业抓住国家级协会大员上前专班推进新引进亿元项目200个以上。实施与央企合作项目38个。组织参加好中博会、厦洽会、高交会等活动办好玉雕节、第十二届张仲景医药文化节、中国月季花展等节会。扎实推进中心城区一体化招商全面实行重大招商项目代办、领办制度提前落实好项目落地支撑保障条件提高开放招商实效。三是持续深化区域合作。把深化京宛对口协作作为一项战略工程强力推进按照“六个一批”的工作要求力争在产业转移、企业嫁接、帮扶结对等方面有大的进展。加快推进中关村科技园、水科技园建设争取更多合作项目入驻。加强与上海张江高科技园区的合作。探索推进与珠三角地区社会经济组织的合作。四是推动外贸转型升级。组织实施科技兴贸战略、品牌发展战略扩大食用菌等农产品出口培育新的出口增长点。推动市级出口基地上档升级。积极创建出口工业产品和出口食品农产品质量安全示范区。引导企业“走出去”发展。

(九)改革创新激活转型发展新引擎

以改革创新破除发展障碍、培育发展动力、增创发展优势让创新创造创业活力在南阳大地充分涌流形成大众创业、万众创新的生动局面。

深入推进各项改革。跟进落实中央、省委部署的重点改革事项着力破解制约发展的突出障碍和人民群众反映强烈的突出问题以政府自身革命推进经济体制改革以经济体制改革引领全面改革加快构建有利于科学发展的体制机制。深化行政体制改革。进一步简政放权、放管结合承接和落实好省下放和取消的审批事项继续取消、下放市级行政审批事项。对重点项目、重大招商项目全面实行联审联批。继续推进政府职能转变和机构改革。分类推进事业单位改革。加大向社会购买公共服务力度。积极推进财税、投融资体制改革。改进预算管理推进各级政府和部门预决算公开。出台实施理顺市区财政管理体制的意见。力争全面完成“营改增”改革。消除投资障碍建立重大项目发布制度吸引社会资本参与教育、医疗、生态环保等领域项目建设。加快推进价格改革。全面放开竞争性领域和环节的商品、服务价格。做好省下放的区域性价格和收费管理。推进水、电、气等资源性产品价格改革。深化国企国资改革。基本完成国有企业改制遗留工作。积极发展混合所有制经济。实行国有资本经营预算制度。探索推进经营性国有资产统一监管。深化农村各项改革。全面完成农村集体土地所有权确权登记发证工作全面铺开农村土地承包经营权确权登记颁证工作基本完成农村宅基地、集体建设用地使用权确权登记颁证工作。深化现代农业综合改革试点工作。推进农村小型水利工程管理体制改革。深化医药卫生体制改革。巩固基层医疗卫生机构改革成果。深入推进县级公立医院综合改革。探索推动城市公立医院改革试点工作。同时加快文化体制、生态环保、社会事业、住房公积金等领域的改革。

强力实施创新驱动。深入推进科技和经济紧密结合推动产学研深度融合加快推进以科技创新为重点的全面创新带动知识创新、服务创新、制度创新、组织创新、管理创新。加快创新能力建设。建好、用好中关村科技园、京宛科技综合服务等科技创新平台。鼓励骨干企业、科研院所建设高水平研发中心争取3家企业被认定为国家高新技术企业新创建一批国家级、省级企业技术中心、工程技术研究中心。加快科技研发和成果转化。组织实施109个重大科技创新项目。加快科研成果、专利技术商品化、产业化。加强质量和品牌建设。围绕建设质量强市坚持质量引领、创新驱动在质量提升中增强创新优势打造品牌南阳。发挥好市长质量奖的激励引导作用。新创建国家级品牌20个、省级品牌100个。

营造大众创业环境。着力营造有利于大众创业的政策环境、制度环境创造统一透明、有序规范的市场竞争环境鼓励自主创业加强创业培训树立创业典型营造鼓励创新创业的浓厚氛围培育壮大南阳经济社会发展的生力军。支持好各类民营经济和中小微企业发展。

加强国防动员、国防教育和国防后备力量建设增强全民国防观念支持驻宛解放军、武警部队和民兵预备役建设力争蝉联全国双拥模范城。广泛开展文明单位、文明镇村等群众性精神文明创建活动。支持工会、共青团、妇联、工商联等人民团体发挥桥梁纽带作用。继续做好外事侨务、对台、民族、宗教、统计、审计、史志、档案、气象、地震、驻外办事机构等工作。

科学编制“十三五”规划加强事关全局的重大战略、重大工程、重要政策措施的研究做好上下衔接协调工作争取一批重大项目列入国家规划。

三、加强政府自身建设

在新常态下推动南阳转型发展、绿色崛起需要倍加努力、不懈奋斗必须加强政府自身建设致力打造法治政府、效能政府、廉洁政府。

(一)坚持依法行政。认真贯彻落实党的十八届四中全会精神强化用法治的思维、手段和方式调节经济运行、管理社会事务严格按照法定权限和程序行使权力、履行职责。坚决执行人大及其常委会决议决定自觉接受监督定期向人大及其常委会报告工作、向政协通报情况认真办理人大代表建议和政协提案广泛听取民主党派、工商联、无党派人士和人民团体意见。完善重大行政决策制度和决策程序。严格落实重大决策终身责任追究制度和责任倒查机制。建立政府法律顾问制度。深化行政执法体制改革推动规范文明执法。加快建立权力清单、责任清单和负面清单让政府法无授权不可为、法定职责必须为让社会法人法无禁止皆可为。

(二)加强效能建设。把落实求效贯穿政府工作始终以踏石留印、抓铁有痕的工作力度以有力有效的工作机制提升政府效能和执行力。实施重大事项、重点项目清单式管理定期向全社会亮进度、晒问题以过程控制、监督倒逼强力推动工作落实。建立部门大统筹、大协同机制实行工作提醒制度形成推动工作的强大合力。全面推进政务公开充分发挥电子政务督查系统作用创造条件让人民监督和批评政府使政府不能懈怠、不敢懈怠。完善科学高效发展绩效考评体系对先进典型大张旗鼓表彰奖励对影响工作、贻误发展的人和事坚决惩处。

(三)持续转变作风。持续巩固拓展教育实践活动成果认真落实中央、省委和市委关于改进作风的有关规定深入开展“一改双优”和“三亮三评”活动建立健全作风建设长效机制。始终牢记、认真践行市委“团结凝聚战斗力敬畏规矩树形象忠诚担当事上见落实见效真本事”的要求。以猛药祛沉疴对长期以来企业和群众反映强烈的效能不高、环境不优的问题以壮士断腕的决心、刮骨疗伤的勇气强力治庸治懒治散严厉惩处不作为、慢作为、乱作为的单位和人员使作风建设的要求真正成为带电的高压线。以零容忍的态度严惩腐败分子。厉行勤俭节约严控一般性支出推动公务用车制度改革确保“三公”经费只减不增以为民务实清廉的作风保障发展、取信于民。

各位代表!改革发展的美好前景鼓舞着我们人民群众过上更好生活的热切期盼鞭策着我们。让我们在市委的坚强领导下振奋精神务实重干团结奋斗圆满完成各项目标任务奋力夺取建设大美南阳、活力南阳、幸福南阳新胜利!
